# Reference data on *in vitro* anatomy and indentation response of tissue layers of musculoskeletal extremities

**DOI:** 10.1038/s41597-020-0358-1

**Published:** 2020-01-15

**Authors:** Tyler Schimmoeller, Erica E. Neumann, Tammy M. Owings, Tara F. Nagle, Robb W. Colbrunn, Benjamin Landis, J. Eric Jelovsek, Tod Hing, Joy P. Ku, Ahmet Erdemir

**Affiliations:** 1 Department of Biomedical Engineering, Cleveland Clinic, Cleveland, Ohio USA; 2 Computational Biomodeling (CoBi) Core, Lerner Research Institute, Cleveland Clinic, Cleveland, Ohio USA; 3 BioRobotics and Mechanical Testing Core, Medical Device Solutions, Lerner Research Institute, Cleveland Clinic, Cleveland, Ohio USA; 4 Department of Obstetrics and Gynecology, Duke University School of Medicine, Durham, North Carolina USA; 5 Department of Bioengineering, Stanford University, Stanford, California USA

**Keywords:** Data acquisition, Musculoskeletal models

## Abstract

The skin, fat, and muscle of the musculoskeletal system provide essential support and protection to the human body. The interaction between individual layers and their composite structure dictate the body’s response during mechanical loading of extremity surfaces. Quantifying such interactions may improve surgical outcomes by enhancing surgical simulations with lifelike tissue characteristics. Recently, a comprehensive tissue thickness and anthropometric database of *in vivo* extremities was acquired using a load sensing instrumented ultrasound to enhance the fidelity of advancing surgical simulations. However detailed anatomy of tissue layers of musculoskeletal extremities was not captured. This study aims to supplement that database with an enhanced dataset of *in vitro* specimens that includes ultrasound imaging supported by motion tracking of the ultrasound probe and two additional full field imaging modalities (magnetic resonance and computed tomography). The additional imaging datasets can be used in conjunction with the ultrasound/force data for more comprehensive modeling of soft tissue mechanics. Researchers can also use the image modalities in isolation if anatomy of legs and arms is needed.

## Abstract (summary)

**Table:** 

Measurement(s)	anthropometric measurement • anatomical structure • tissue section thickness • indentation response
Technology Type(s)	anthropometric tape • magnetic resonance imaging (MRI) • computed tomography • ultrasound imaging • force transducer
Factor Type(s)	age • mass • sex • experimental condition • height • race
Sample Characteristic - Organism	Homo sapiens

Machine-accessible metadata file describing the reported data: 10.6084/m9.figshare.11494626

## Background & Summary

The musculoskeletal system is a multi-layer support and protective structure composed of skin, fat and muscle. The tissue properties of individual layers and composite structure influence the musculoskeletal system’s ability to respond to mechanical loading, in some cases, loads that may result in injury. Extremities are prone to injuries that range from gun-shot wounds or blast injuries^1,2^ to minor cuts and bruises and require surgical intervention depending on severity. High fidelity surgical simulations can enhance surgical training by representing real tissue characteristics.

The past two decades have seen significant advancements in virtual reality haptic feedback simulations that aim to increase the effectiveness of surgical training^3^, and long before that, soft tissue characterization was possible using continuum mechanics techniques^4,5^. However, a foundational database containing accurate tissue thickness and mechanical properties did not exist until recently^6^. To obtain accurate tissue thickness and mechanical properties, non-destructive techniques have been used, e.g., imaging. Ultrasound is a cost-effective and non-invasive imaging modality best used to investigate soft tissue properties. If used in conjunction with force feedback the user can account for the applied load deforming tissue during imaging^7^. Computer tomography (CT) and magnetic resonance (MR) imaging are also used to investigate soft tissue properties and, although more cost prohibitive, allow accurate measurement of tissue thickness. These imaging modalities also have the added benefit of being three-dimensional (3D) image volumes and can be segmented to reconstruct detailed 3D anatomical models.

In a recent and similar study completed by the authors^6^, instrumented ultrasound and anthropometric measurements were performed to quantify multi-layer and aggregate tissue thickness as well as characterize the mechanical properties of the musculoskeletal extremities *in vivo*. The goal of the current study was to expand and improve existing datasets by performing more extensive experimentation *in vitro*. Additional imaging modalities were included to allow tissue thickness comparison or individual analyses by implementing methods of spatial association via implanted fiducial markers.

A related dataset^8,9^ examined surgical tool kinetics and kinematics as well as surface strain measurements using the same specimens from the study presented in this data descriptor. Briefly, three instrumented surgical tools, including a scalpel, retractor and forceps, as well as an indenter, were used to perform seventeen different surgical acts on the skin, fat, and muscle layers of cadaveric legs. The data collected includes forces acting at the tool tip and motions of the tool with respect to the specimen. Surface strain measurements were also captured for indentation, cutting, and pinching surgical acts.

## Methods

### Specimen overview

The right arm and leg of nine cadaver specimens (each set from the same donor) were obtained with the approval the Human Research Protection Office of the U.S. Army. The donors consisted of four females and five males with an average age of 51.6 years (range 39–65 years) and an average body mass index (BMI) of 23 (range 19–25). Exclusion criteria included any past injuries or surgeries performed on extremities, as well as having a BMI outside the range of 18 to 29. The criteria limited adverse visual artifacts and ensured the full depth of soft tissue to be imaged via ultrasound, which, unlike MR and CT imaging, has depth limitations.

### Experimentation workflow

**Fig. 1:**
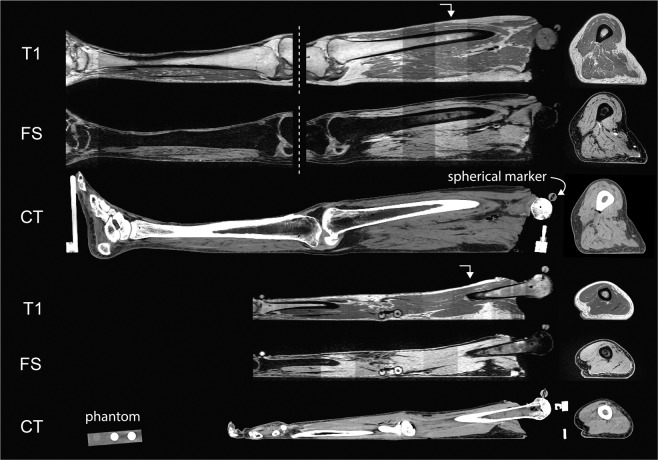
All CT and MR imaging modalities of both leg (top 3 rows) and arm (bottom 3 rows). Row labels are as follows: T1: T1 weighted magnetic resonance, FS: T1 weighted magnetic resonance with fat saturation, CT: computed tomography. The sagittal cross sections are shown to the left (viewing from medial to lateral), and axial to the furthest right (viewing from inferior to superior). Directional arrows show where the axial sections were taken along the length of the sagittal plane. Note the leg MR images were separated into upper and lower segments due to the limited field of view of MR machines. Also shown in the fourth row (CT arm) is an axial cross section of a phantom object used in all CT scans.

**Fig. 2:**
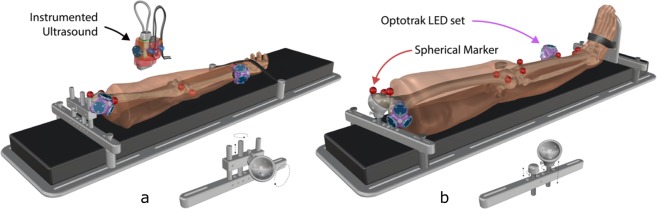
The custom fixture used for imaging. Two variations are shown, (**a**) with arm attachments and (**b**) with leg attachments. Each is fully adjustable to accommodate anatomical differences in a variety of specimens. The foot and hand were secured with Velcro, while the femoral and humeral heads were screwed in to a 3D printed receiver with nylon screws. All fixture materials were non-metal to reduce imaging artifacts in CT and MRI. Also shown are the instrumented ultrasound and Optotrak LED triads.

Experimentation started with specimen preparation, followed by CT imaging (Fig. 1), and finally MR imaging (Fig. 1). The specimen was then stored in a custom-made fixture (Fig. 2) in a walk-in refrigerator overnight due to the time required for imaging. The next day, anthropometric measurements were recorded followed by ultrasound imaging.

### Specimen preparation

**Fig. 3:**
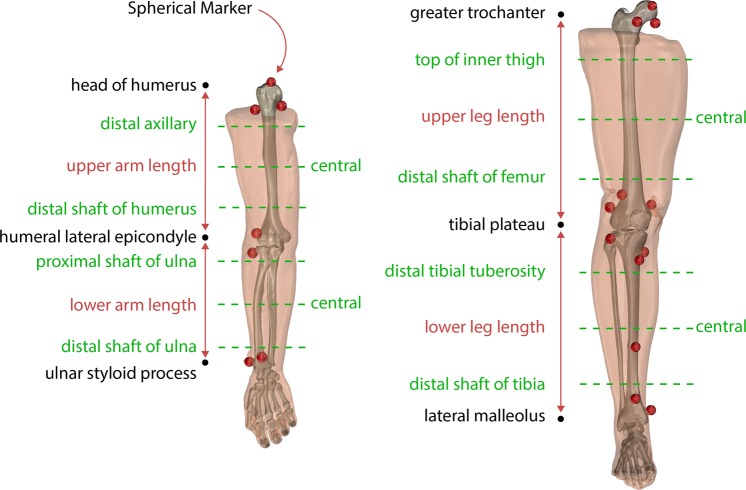
Arm and leg anthropometric measurement sites displayed on a rendering of an arm and a leg. Circumference measurements were taken at each dashed green line. Each ultrasound image was taken on the anterior, posterior, medial, and lateral positions around the circumference of each dashed line. Also shown are the implanted spherical markers for registration (red).

When specimens were received, they were kept in a freezer at −20 °C and removed twenty-four hours prior to experimentation. A custom-made fixture (Fig. 2) was used to support the specimen and maintain consistent and neutral anatomical position throughout testing and transportation. The disarticulated limbs did not naturally rest in anatomical supine position, so the femoral and humoral heads were rigidly attached to height adjustable 3D printed mounts on the fixture. Additionally, the foot and hand were secured to further limit movement during transportation and imaging. The leg fixture also included a height adjustable rod for the greater trochanter so that it would rest lower than the femoral head, much like the typical anatomical supine position. Each leg and arm were equipped with hollow, 3D printed, MR and CT visible spherical markers (see Figs. 1–3) having a 10.0 mm radius and secured with nylon screws. The leg had twelve markers (six on the femur and six on the tibia) and the arm had seven markers (four on the humerus and three on the radius) (Fig. 3). The number of markers used in each was limited by the size of the humerus and radius as well as the desire for not disrupting the region of interest. A minimum of three markers was needed to establish a coordinate system, but more were used to reduce registration errors between imaging data and motion tracking system.

The specimens were equipped with two sets of smart cluster triads composed of nine LEDs each, by Optotrak Certus (Northern Digital Inc., Waterloo, Ontario) after CT and MR imaging. For the leg, the triads were placed at the femoral head and on the mid-shaft of the tibia (Fig. 2). For the arm, the triads were placed on the humeral head and distal end of the radius (Fig. 2). Placement of the triads was chosen to limit interference with desired ultrasound locations while remaining visible to the Optotrak camera system during experimentation.

### CT and MR image acquisition

Both CT and MR protocols were selected to enhance soft tissue clarity and tissue differentiation of the skin, fat, and muscle layers. CT imaging was conducted at the Cleveland Clinic, Cleveland, Ohio using a SOMATOM Definition Edge 128 scanner (Siemens Healthcare, Erlangen, Germany) with a 0.6 mm slice thickness and no gap using a B40 reconstruction kernel. A phantom was placed in the region of interest with three rods (0, 500, and 1000 mg HA/cm^3^). The axial in-plane resolution ranged from 0.51 mm to 0.59 mm depending on the reconstruction size of the area of interest. The entire specimen i.e., upper and lower appendage, was scanned in a single pass.

MR imaging was conducted at University Hospitals, Cleveland, OH using a 3T Skyra (Siemens Healthcare, Erlangen, Germany) with a 2 mm slice thickness, 1 mm gap, and an axial in-plane resolution of 0.5 mm. Two imaging protocols were used; T1 weighted without fat saturation and T1 weighted with fat saturation (FS). The repetition time (TR) and echo time (TE) were 500 ms and 11 ms respectively. The specimens were scanned by segment i.e. upper/lower arm/leg in twenty-five slice increments due to the limited field of view of MR imaging with the desired slice thicknesses. Each segment was scanned twice before moving to next section to include both T1 weighted and T1 weighted with FS. Depending on the size of the specimen, each segment was scanned in four to five sections. The specimen was not moved between section scans to maintain alignment in a single imaging coordinate system. A custom Python script (http://www.python.org) concatenated the sections automatically so that the each segment could be viewed as one continuous scan.

### Anthropometric measurements

Circumference and length measurements were recorded for each specimen using a soft tape measure. Each appendage was nominally divided into an upper and lower segment, e.g. the upper arm was elbow to humoral head. At each segment, the circumference and the distance to the most superior landmark were recorded at the proximal, middle, and distal locations in addition to the total length (Fig. 3). The landmarks used were identical to our previous *in vivo* study^6^ with exception to the inferior lateral acromion process, where the current study used the head of the humerus (the scapula was removed prior to experimentation).

### Ultrasound image acquisition

Ultrasound imaging was performed using an ACUSON S3000^TM^ (Siemens Healthcare, Erlangen, Germany), instrumented^7^ with a six degree-of-freedom (6-DoF) Nano25 load cell (ATI Industrial Automation, Apex, North Carolina), a VN-100 inertial measurement unit (IMU) (VectorNav, Dallas, Texas), and an optical tracking smart cluster (Northern Digital Inc., Waterloo, ON) (Fig. 2). Depending on the depth of tissue to be imaged, a 9L4 transducer (head dimensions 45 mm × 15 mm) or 14L5 transducer (head dimensions 45 mm × 8 mm) was used. The probe used for each trial is identified in the trial specific state configuration file.

A gravity compensation algorithm was used in a custom program based on simVITRO^®^ LabVIEW packages (Cleveland Clinic, Cleveland, Ohio) to offset the weight of the ultrasound probe in any orientation. The attached IMU and load cell first measured the probe weight in mid-air, in six orientations, to determine a spatial relationship between probe weight measured by the load cell and orientation. The weight of the probe in any orientation became a function of orientation, therefore in any orientation the probe weight could be subtracted. This method of weight compensation was previously used and validated^6,7^.

Just prior to ultrasound imaging, the 3D printed spherical markers on the specimen were digitized in the motion capture coordinate system to spatially relate the optically tracked ultrasound to the imaging coordinate systems. In addition, a bone coordinate system was setup for each segment, by digitizing bony landmarks (Table 1), to spatially relate the optically tracked ultrasound and digitized points to the relevant bone position. The upper and lower appendages were treated as separate rigid bodies.

**Table 1:** Bony fiducial markers used create a bone coordinate system from the global motion capture coordinate system.

Extremity	Fiducial Marker
Upper Leg	Lateral Femoral Epicondyle
	Medial Femoral Epicondyle
	Femoral Head (4 pts)
Lower Leg	Lateral Tibial Plateau
	Medial Tibial Plateau
	Medial malleolus (2 pts)
	Lateral malleolus (2 pts)
Upper Arm	Lateral Humeral Epicondyle
	Medial Humeral Epicondyle
	Humeral Head (4 points)
Lower Arm	Lateral Humeral Epicondyle*
	Medial Humeral Epicondyle*
	Ulna Styloid

Note: Lower Arm coordinate system established using two points from Upper Arm.

**Fig. 4:**
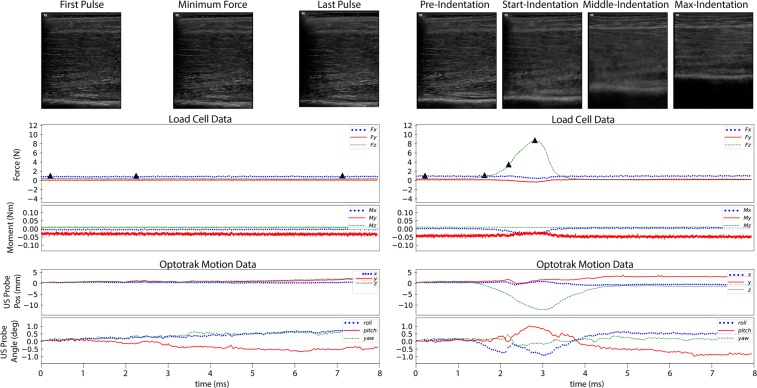
Sample load and motion data. The left panel shows an anatomical trial (032_CMULTIS012-1_UL_PC_A-1) and the right panel shows an indentation trial (033_CMULTIS012-1_UL_PC_I-1). From top to bottom are ultrasound images, forces, moments, and motion of ultrasound probe (position and angle). The forces and moments were reported in a probe tip coordinate system originating on the center face of the ultrasound probe (the Z-axis is aligned perpendicular to the face and in the direction of applied load). The motion was reported in global motion capture coordinate system. The triangle shapes on the force plot represent the time instance in which the respective ultrasound image was taken.

Soft tissue thicknesses and indentation response were captured through freehand anatomical ultrasound imaging and indentation ultrasound imaging, respectively. Anatomical ultrasound imaging (Fig. 4) was performed at the anterior, posterior, medial, and lateral positions at proximal, middle, and distal regions of each upper and lower leg and arm, totaling 24 per appendage. The applied load during anatomical imaging was minimized to limit soft tissue deformation and was considered minimal if the measured load was less than 2N; measured by the attached load cell. Indentation ultrasound imaging (Fig. 4) was performed at four locations per arm and per leg, the central anterior and central posterior region of each segment. The indentation trials were performed immediately following the respective force trials of the same location to ensure minimal changes in location. It is important to note that the loading rate of the ultrasound probe was not controlled, due to the freehand nature of ultrasound imaging. A limitation of this dataset is the lack of indentation response collected at varying loading rates. While this data may be important to capture the full mechanical behavior of the soft tissue indentation response, the data included in this dataset provides a realistic field assessment of the indentation response without the need for a sophisticated loading mechanism.

The total time of each image acquisition was set to eight seconds. Once the target region e.g. upper arm was located, the probe was oriented so that the target bone (region dependent, shown in Table 2) was visible. The long axis of the probe was oriented along the bone, therefore, the bone appeared as a flat and bright landmark spanning the width of the image. The depth, gain, and focus were adjusted so that the bone was visible and the multi-layer delineation between skin, fat, and muscle was as clear as possible.

**Table 2:** All ultrasound bone targets per limb segment.

Extremity	Positions	Targeted Bone
Upper Arm	All	Humerus
Lower Arm	Anterior, Posterior, Lateral	Radius
	Medial	Ulna
Upper Leg	All	Femur
Lower Leg	Anterior, Lateral, Medial	Fibula
	Posterior	Tibia

The specimen remained in its fixture for accessing the medial, anterior and lateral positions. It was then rotated such that the posterior faced upwards to reveal six posterior imaging locations.

### Spatial alignment

The spherical markers, which can be seen in Fig. 3, were used to establish a relationship between the coordinate system of motion capture system, bone coordinate system, ultrasound acquisition and the anatomical imaging coordinate systems (CT and MR). The boundaries of the spheres were extracted from the MR and CT images using image segmentation (3D Slicer^10^ – http://www.slicer.org), resulting in STL surfaces in each image coordinate system. A custom Python script was used to perform the spatial alignment for each segment between the various coordinate systems, i.e. digitized points from bone coordinate system (DG) to CT, DG to MR, and MR to CT. First, the digitized points on the spheres were transformed from the global motion capture coordinate system (set by the camera) to the appropriate bone coordinate system (where the origin is at the knee or elbow). A sphere was fit to both the STL surfaces and the digitized points and a transformation matrix was established between the bone coordinate system and the image coordinate system using the singular value decomposition method^11^. This transformation matrix, along with the others saved during data collection, allow the user to examine the data (positions, forces/moments, images) in their desired coordinate system. The alignment allows users to compare images in the same location across all three modalities. As previously mentioned, the appendages were nominally divided in to upper and lower segments. Each segment was treated as a rigid body and each segment had its own set of spherical markers. The project wiki provides additional details on the workflow of the spatial alignment process (https://simtk.org/plugins/moinmoin/multis/Specifications/DataAnalysis/Registration).

### Thickness analysis

The multi-layer tissue thicknesses were determined using previously established ultrasound thickness measurement methods^6^. Briefly, the graphic user interface was used to display the ultrasound frame(s) at the minimum applied force for anatomical trials and throughout the compression portion of the indentation trials. In each image, moveable red dots were placed at each of the boundaries to represent skin, fat, and muscle thicknesses. The script was modified to store ultrasound position and orientation, in addition to the forces, moments, and thicknesses for each frame in an XML format. Soft tissue mechanics may be extracted from the summarized data, using the deformation defined by the thickness change during indentation and the applied force. In addition, the experiment motion may be re-created virtually or physically using the position and orientation data of the ultrasound probe in relation to the limb.

## Data Records

A set of naming conventions was created to help organize and later navigate all raw and derivative data^12^. Each de-identified donor was given a unique ID and identical folder structure. It should be noted that the structure is nearly identical in name and description as the *in vivo* reference data^6^ with several additional folders and modifications to descriptions to reflect the *in vitro* data. An asterisk (*) denotes an addition or modification to the format of the *in vivo* data structure.

### Raw data

1. Data* – Probe load and orientation data in raw and transformed (ultrasound probe tip coordinate system with weight compensation) representations. Additionally, Optotrak motion data was included (.tdms). For clarification, this directory was taken directly from the data collection software, however it does not contain all forms of data collected within this dataset (see additional directories below). It was not re-named to avoid manual data entry errors.
2. *Configuration* – Contains the sensor and state files used during data collection for each data file (.cfg). The sensor and state files provide the structure and description of the binary data file (.tdms) for the raw and transformed data, respectively. Also contains the subject configuration file [.cfg and .xml, which provides the file locations (state.cfg, sensor.cfg, data.tdms) of each data collection site and de-identified subject information (demographics in Table 3 and anthropometrics)].

**Table 3:** A key for demographics recorded per donor in each Donor XML file.

Data Category	Description	Key
Age	Donor’s age in years	N/A
Gender	Donor’s gender	0 – Male
		1 – Female
Ethnicity	Donor’s ethnicity	0 – Hispanic or Latino
		1 – Not Hispanic or Latino
Race	Donor’s race	0 – White
		1 – Black or African American
		2 – American Indian or Alaska Native
		3 – Asian
		4 – Native Hawaiian or Other Pacific Islander
Height	Donor’s height in centimeters	N/A
Mass	Donor’s mass in kilograms	N/A
3. *Ultrasound* – DICOM image volumes for each trial (.IMA)
4. CT* – Computed tomography dataset of an arm and leg presented in a NIfTI file format. The full leg and arm were scanned in one pass, therefore a single volume represents an entire appendage (.nii).
5. MRI* – Magnetic resonance imaging set for an upper and lower arm and leg (four files). Each segment, e.g. upper arm, was composed of multiple smaller sections (25 images per section) stitched together to create a composite NIfTI file using a custom Python script (.nii). Sections are visible by their subtle difference in contrast.

### Derivative data

File association:

1. DataOverview* – Provides a summary figure of each individual trial, including ultrasound frames throughout the trial with corresponding forces and moments in the ultrasound probe tip coordinate system. Additionally, this figure includes six plots depicting Optotrak motion (translation and rotation) of the bone, ultrasound probe, and ultrasound probe relative to bone for each trial.
2. *FileAssociation* – Provides a figure for each individual trial showing the raw and time adjusted analog signal between the data (.tdms) and ultrasound (.IMA) systems for time synchronization.
3. *TimeSynchronization* – Contains an XML for each trial that stores the time shift (dT, measured in milliseconds) between the data (.tdms) and ultrasound (.IMA) systems.

Spatial alignment:

- *Marker STL’s –* Segmented spherical markers of both CT and MR used to align limb segments (STL)
- *Quality Check –* The distances between each marker, radius of each marker, and summary statistics provided in a text document (TXT)
- *Ultrasound Positions –* Provides the ultrasound location in CT and MR for each ultrasound trial in the form of a visual toolkit file. This can be used with the provided Python scripts to show the CT or MR image in the same location at the same angle as recorded by ultrasound (XML).

Thickness analysis:

- *TissueThickness* – Sub-directory that contains all thickness analysis.
- *UltrasoundManual –* Manual identification of tissue boundaries from ultrasound imaging of each trial (XML)
- *ThicknessPNG –* Provides image of the first ultrasound frame that was analyzed (corresponding to minimum force or initiation of indentation, for anatomical and indentation trials, respectively) and a plot of force magnitude vs. thickness (skin, fat, muscle, and total).

Raw and derivative data files exist for each trial with the following root naming convention: *“Experiment Run Number”_”Donor ID”_”Limb Segment”_”Location”_”Test Type”-”Trial Number Index”*

- Experiment Run Number: 3-digit number that gets auto-incremented for each tdms file (order in which data are collected for each specimen).
- Donor ID: 3-digit number that gets assigned to each donor in the order that they were acquired.
- Limb Segment: Two letter abbreviation for the trial segment, UA = upper arm, LA = lower arm, UL = upper leg, LL = lower leg,
- Location: Two letter abbreviation describing the imaging location. First letter – (A)nterior, (P)osterior, (M)edial, (L)ateral. Second letter – (P)roximal, (C)entral, (D)istal.
- Test Type: I = indentation, A = anatomy
- Trial Number Index: auto-incremented for each trial, i.e. if the location is tested more than once the subsequent data files will be -2, -3, etc.

Additional characters are appended to the root name for further data descriptions. Example file names for a single trial are included in Table 4.

**Table 4:** Example filenames for raw and derivative data.

Data File Description	Example File Name
Donor XML	CMULTIS012-1.**xml**
Sensor configuration	010_CMULTIS012-1_UA_MD_A-1**_Sensor.cfg**
State configuration	010_CMULTIS012-1_UA_MD_A-1**_State.cfg**
Data	010_CMULTIS012-1_UA_MD_A-1**.tdms**
Ultrasound	010_CMULTIS012.**US.MSK.“Date&Time”.IMA**
Data overview	010_CMULTIS012-1_UA_MD_A-1**_analysis.png**
File association	010_CMULTIS012-1_UA_MD_A-1**.png**
Time synchronization	010_CMULTIS012-1_UA_MD_A-1**_dT.xml**
Tissue thickness (xml)	010_CMULTIS012-1_UA_MD_A-1**_manThick“timestamp”.xml**
Tissue thickness (first image)	010_CMULTIS012-1_UA_MD_A-1**_manThick“timestamp”.png**
Tissue thickness (graph)	010_CMULTIS012-1_UA_MD_A-1**_manThick“timestamp”Graph.png**

CT and MR imaging files use the following naming convention: *“Donor ID”_”Limb_Segment”_” Modality”.nii*

- Donor ID: identical to previous description of Donor ID
- Limb Segment: An abbreviation indicating portion of appendage imaged. The newly introduced abbreviations are, WA = Whole Arm, WL = Whole Leg. The remaining abbreviations are identical to the previous Limb Segment descriptors.
- Modality: CT, MRI_FS, MRI_T1 where FS is fat saturation with T1 protocol and T1 is the T1 protocol without fat saturation

Registration files used the following naming convention. In order are the conventions for Marker STL’s, Quality Check, and Ultrasound Positions. The Registration Number and Donor ID are the same for each. Only new conventions are described.

Registration filename prepend: *“Registration Number”_“Donor ID”_*

- Registration Number: If multiple registrations were performed, the naming convention easily allows it
  Marker STL filename postpend: *_”Marker Location”_”Modality”.stl*
- Marker Location: Location of 3D printed spherical markers, F = Femur, T = Tibia, H = Humerus, R = Radius
  Quality Check filename postpend: *_”Modality Comparison”_”Limb Segment”.txt*
- Modality Comparison: CT and MR were previously defined, MQL = Marker Quality Check, DG = Digitized Points from ultrasound motion tracking
  Ultrasound Positions filename postpend: *_”Limb Segment”_”US”_”Modality”.xml*
- US: Ultrasound

The files used for spatial alignment are found in a Registration folder. Example file names for a single donor are included in Table 5.

**Table 5:** Example filenames for spatial alignment data.

Data File Description	Example File Name
Marker STL	R01_CMULTIS012-1_**F1_CT.stl**
Quality Check	R01_CMULTIS012-1_**MQC_DG_CT_LA.txt**
Ultrasound Positions	R01_CMULTIS012-1_**LA_US_CT.xml**

The project website (https://simtk.org/projects/multis) contains all specifications as well as experiment notes. The related dataset^8^ on surgical tool forces is also described on the project website.

## Technical Validation

The ultrasound images, load cell forces, and Optotrak motion tracking represent three separate instruments that must be temporally synchronized to be of significant use. The temporal synchronization of force and ultrasound imaging was established using a custom analog signal routed through each system^7^. Further validation was required to ensure temporal synchronization between the Optotrak motion and instrumented ultrasound system. The motion data was routed through its native software, NDI First Principles (Northern Digital Inc., Waterloo, Onterio), to the custom LabVIEW (National Instruments, Dallas, Texas) program used to record all force and orientation data. A tap test was performed to determine the temporal difference between force and motion. The load cell was physically and instantaneously tapped with enough force to move the instrumented ultrasound system, thereby causing a noticeable change in motion and force. A custom Python script was developed to find the exact time each spike in motion and force occurred. The mean of three trials was 43.0 +/− 16.3 ms. The force was measured with a sampling rate of 1,000 Hz and motion roughly 33 Hz, therefore the error was expected to be close to that of the lower rate (33 Hz or 30 ms).

The error of spatial alignment in each limb segment was analyzed by calculating the vector between all pairs of markers (e.g. the lower arm pairs include R1 to R2, R1 to R3, and R2 to R3). The distance (magnitude of the vector) was compared between each coordinate system, i.e. DG to MR, DG to CT, and MR to CT. In addition, the angle difference was calculated using the dot product between the line segments in each coordinate system. The average distance differences as well as angles across all thirty-six segments are presented in Table 6.

**Table 6:** The mean and standard deviation of distance differences, percentage differences, and angle differences when comparing the thicknesses measured in the bone coordinate system, CT and MR via the segmented spherical markers.

	DGCT	DGMR	MRCT
distance diff.	0.64 +/− 0.56	1.12 +/− 0.78	1.12 +/− 0.84
percentage diff.	0.52 +/− 0.76	0.93 +/− 0.99	0.76 +/− 0.73
angle diff.	0.34 +/− 0.53	0.46 +/− 0.61	0.29 +/− 0.39

Note: DGCT = digitized points to CT. DGMR = digitized points to MR. MRCT = MR to CT.

The thickness analysis methods, including software and observers, were identical to the authors previous work^6^. In the previous study, repeatability was evaluated using all anatomical and indentation trials from 5 random patients and the inter- and intra-observer variability was measured using two observers. The inter-observer mean and standard deviation thickness measurements for anatomical trials was 0.36 ± 0.38, 0.78 ± 1.10, 0.65 ± 1.11, and 0.32 ± 0.38 mm for skin, fat, muscle, and total tissue thickness respectively. Likewise, the intra-observer was 0.16 ± 0.17, 0.49 ± 1.19, 0.48 ± 1.19, and 0.19 ± 0.19 mm. The thickness change during indentation was measured from the beginning of indentation to maximum force. The inter-observer mean and standard deviation thickness change for skin, fat, muscle, and total thickness was 0.15 ± 0.19, 0.29 ± 0.32, 0.31 ± 0.31, and 0.42 ± 0.37 mm, respectively. Likewise, the intra-observer mean and standard deviation was 0.10 ± 0.12, 0.24 ± 0.24, 0.32 ± 0.25, and 0.29 ± 0.23 mm.

## Usage Notes

All data can be found on the active working version of the data management site: https://multisgamma.stanford.edu/. A static version of the complete dataset is also provided^12^.

More detailed specifications including but not limited to Python scripts, hardware integration and design, the custom LabVIEW program and more can be found on the project website (https://simtk.org/plugins/moinmoin/multis/). A source code repository with downloadable scripts and data can be found at https://simtk.org/scm/?group_id=1032.

## Data Availability

The source code repository is available at (https://simtk.org/projects/multis). Additionally, two static packages containing thickness analysis software and all data/source code used in the technical validation section are available (https://simtk.org/frs/?group_id=1032). In order to use the provided scripts, one needs to install Python 2.7 with Anaconda (https://docs.anaconda.com/anaconda/). The two additional libraries required to read ultrasound and data files respectively are DICOM and npTDMS. Additional libraries used in the provided scripts can be installed using Anaconda (“conda install” command).
